# The nasopharyngeal carcinoma in Xiamen China from 2011 to 2020: a population-based linkage study

**DOI:** 10.1186/s12885-025-15394-0

**Published:** 2025-12-08

**Authors:** Jiali Quan, Aiping Zhou, Zhinan Guo, Youlan Chen, Lingxian Qiu, Jiahuang Chi, Yue Huang, Yilan Lin

**Affiliations:** 1 Xiamen Center for Disease Control and Prevention, Xiamen, Fujian China; 2 State Key Laboratory of Vaccines for Infectious Diseases, Xiang an Biomedicine Laboratory, School of Public Health, Xiamen University, Xiamen, Fujian China; 3 Nanjing DrumTower Hospital, Affiliated Hospital of MedicalSchool, Nanjing University, Nanjing, Jiangsu China; 4 School of government, Nanjing University, Nanjing, Jiangsu China; 5 Xiamen Medical College, Xiamen, Fujian China

**Keywords:** Nasopharyngeal carcinoma, Incidence, Mortality, Survival rate, Trend

## Abstract

**Background:**

Nasopharyngeal carcinoma (NPC) is not common in most parts of the world but is particularly prevalent in southern China. This study analyzed NPC incidence, mortality, years of life lost (YLL), and survival rates in Xiamen from 2011 to 2020.

**Methods:**

This study mainly utilized data from the Xiamen City Cancer Registry, and cancer follow-up cohort, covering the period from 2011 to 2020. Crude and age-standardized incidence, mortality, YLL, and survival rates, as well as their trends were analyzed using annual percent change (APC) and average annual percent change (AAPC), stratified by sex and residency status.

**Results:**

From 2011 to 2020, Xiamen reported 996 new NPC cases (733 males, 263 females) and 513 deaths (396 males, 117 females). Age-standardized incidence, mortality, and YLL rates were 3.58/100,000, 1.83/100,000, and 65.10/100,000, respectively. The AAPC in incidence and mortality rates of NPC was − 5.48% (95% CI: −9.25, −1.54, *P* < 0.05) and − 0.82% (95% CI: −6.01, 4.65, *P* > 0.05), respectively. The 5-year age-standardized relative survival rate was 55.98% (95%CI: 52.08, 60.17).

**Conclusion:**

Over the recent decade, a consistent decline in the incidence of NPC has been observed, accompanied an insignificant decreasing mortality trend in Xiamen. Future efforts could focus on enhancing prevention, screening, and treatment strategies to potentially reinforcing these positive trends.

**Supplementary Information:**

The online version contains supplementary material available at 10.1186/s12885-025-15394-0.

## Introduction

Nasopharyngeal carcinoma (NPC) is an aggressive malignancy of the head and neck, originating in the outermost epithelial layer of the nasopharyngeal cavity. This region is situated above the oropharynx and hypopharynx, closely adjacent to the base of the skull [1]. According to the International Agency for Research on Cancer (IARC), the year 2022 recorded approximately 120,434 new cases of NPC and 73,482 associated fatalities [2]. Most of these cases (>80%) were confined to countries in Asia. NPC exhibits a high incidence particularly in China, across provinces spanning from the eastern to the southeastern regions. Notably, areas such as Guangdong, Guangxi and Hong Kong exhibit an age-standardized incidence rate (ASIR) exceeding 10 cases per 100,000 population [3]. Other provinces, such as Zhejiang, Fujian, and Hunan are typically considered to be regions of intermediate risk (ASIR ranges from 3 to 5 per 100,000). Conversely, in the northern and western regions, NPC is deemed an uncommon cancer with an ASIR of less than 2 per 100,000 [4]. In 2022, the ASIR of NPC in China was 2.4 per 100,000, with males exhibiting a 2.6-fold higher rate than females (3.4 vs. 1.3 per 100,000) [2].

Over the past decades, NPC incidence has declined gradually worldwide: substantial reductions have been observed in South and East Asia, North America, and the Nordic countries, with the average annual decrease ranging from 1% to 5%. This decline is likely attributed to lifestyle and environmental changes, enhanced understanding of the pathogenesis and risk factors, population screening programs, advancements in diagnostic techniques, and individualized comprehensive chemoradiotherapy strategies [1]. Previous studies have documented a decline in the incidence of NPC in Hong Kong over the past decade, with the average annual changes of −1.9% for males and − 4.3% for females between 2010 and 2019 [5]. Bai et al. reported that over the last three decades, the age-standardized mortality rate (ASMR) and crude mortality rate (CMR) for NPC in China have decreased, whereas the ASIR and crude incidence rate (CIR) have increased [6]. The trend of NPC incidence and mortality vary significantly across different countries and even among regions within the same country. Understanding the region-specific epidemiology and long-term trends of NPC is crucial for informing evidence-driven policies for NPC control. Current scholarly investigations are primarily concentrated on high-risk region, including Guangdong and Guangxi [7, 8]. Few studies have comprehensively reported the data in other regions. This study aimed to investigate the epidemiological characteristics of NPC in Xiamen, a typical intermediate-risk region, to assess the trend of incidence, mortality, years of life lost (YLL) and survival rates in the region and provide a reference for allocating health resources and planning future health policies.

## Materials and methods

### Data sources

Data were collected from the Xiamen Cancer Register, the Xiamen Death Register, household registration, and the cancer follow-up cohort, from 2011 to 2020. Established in 2009, the population-based Xiamen City Cancer Register includes over 3.02 million people across both urban and rural districts, ensuring comprehensive coverage since 2010. Primary cancer-related deaths, recorded annually in the Xiamen Death Register, were used to identify unregistered cancer cases and outcomes, enhancing the register’s accuracy. The household registration system provided demographic information for the covered population.

Survival outcomes were monitored through both active and passive follow-up, with death data sourced from the Xiamen Death Register System and living patients followed via periodic phone calls by community-based family physicians or public health professionals. Survival outcomes and sociodemographic data were also collected. Loss to follow-up was defined as the inability to contact an individual for three consecutive years. The data for our study were updated until September 30, 2023. Before we accessed the information, registry staff had already anonymized the records. We used C11 codes from International Classification of Diseases (ICD-10) to classify NPC cases. The Xiamen City Center for Disease Control and Prevention’s ethics committee approved (XJK/LLSC (2023)004).

### Data quality

The quality of the cancer registry data was evaluated according to the Guidelines for Chinese Cancer Registration and the standards set by the IARC. The quality metrics included the percentage of cases based death certificate only (DCO%), morphological verification rate (MV%), and the mortality-to-incidence ratio (M/I). For NPC, our study reported DCO% at 3.11% (31/996), MV% at 73.09% (728/996), and M/I at 51.51% (513/996), satisfying all established quality criteria.

### Statistical analysis

In this study, the 2011–2020 NPC incidence, mortality, YLL and survival data were statistically analyzed by region, sex and age. Trends in cancer incidence, mortality, YLL and survival were analyzed using the Annual Percent Change (APC) and the Average Annual Percent Change (AAPC). Both incidence and mortality rates were standardized using Segi’s world standard population. YLL is a measure of premature death, calculated by the number of deaths at each age multiplied by a standard life expectancy at that age group. We derived YLL using life expectancy data from the Global Burden of Disease Study 2019 (GBD 2019) reference life table. Total YLLs were the sum of YLL at each age group. Period analysis was used to deal with the censored data. Survival assessments included observed survival rate (OS), relative survival rate (RS), and age-standardized relative survival rate (ARS). Patients with multiple primary tumors or DCO cases were excluded from the survival analysis. The loss-to-follow-up rate was 1.31% (13/996). We refined abridged life tables into complete ones using the Elandt-Johnson model, with OS estimated via the life table method. RS was determined by comparing the OS against expected survival from the general population, following the Ederer II method. ARS was calculated with the standard weights according to the International Cancer Survival Standards (ICCS): 28% for 0–44 years, 17% for 45–54 years, 21% for 55–64 years, 20% for 65–74 years, and 14% for 75–99 years.

The Joinpoint Regression Program (version 5.0.2) was used to calculate the APC and AAPC for trends in NPC incidence, mortality, YLL, and survival rates, while all other analyses were performed using SAS (version 9.4).

## Results

### Incidence trends analysis

**Fig. 1:**
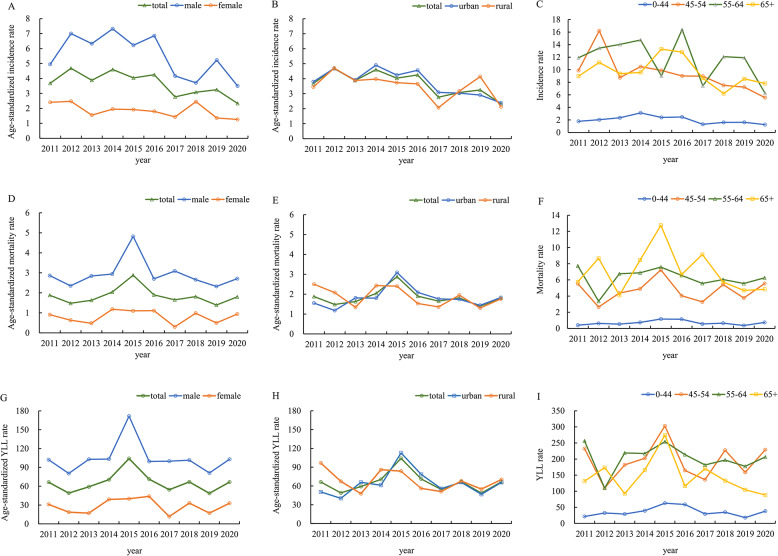
The incidence, mortality, and years of life lost rates of nasopharyngeal carcinoma from 2011 to 2020 in Xiamen. All rates are expressed per 100,000 population

A total of 996 cases of NPC were registered between 1 st January 2011 and 31 st December 2020, with a CIR of 4.61 per 100,000 and an ASIR of 3.58 per 100,000 (Table 1). As shown in Fig. 1; Table 1, there was a higher incidence rate in males compared to females (ASIR 5.42 per 100,000 vs. 1.83 per 100,000), and similar incidence rate between urban residents and rural residents (ASIR 3.66 per 100,000 vs. 3.44 per 100,000). The CIR was low among individuals under 45 years old (1.96 per 100,000) but increased with age, reaching 11.51 per 100,000 for those aged 55–64 years. The cumulative incidence rate from birth to 74 years old was 0.4%, whereas the calculation of incidence rate over the truncated age range of 35–64 years was 8.11 per 100,000 (Table S1). All rates are expressed per 100,000

**Table 1:** The incidence, mortality, and 5-year survival rate of nasopharyngeal carcinoma in Xiamen city from 2011 to 2020

Variable	Incidence					Mortality				5-year survival rate				
	N	CIR	AAPC (95%CI)	ASIR	AAPC (95%CI)	N	CMR	AAPC (95%CI)	ASMR	AAPC (95%CI)	OS (95%CI)	RS (95%CI)	ARS (95%CI)	AAPC^b^ (95%CI)
Total	996	4.61	−4.16^c^ (−11.91, 4.27)	3.58	−5.48^*^ (−9.25, −1.54)	513	2.37	0.39 (−7.58, 9.05)	1.83	−0.82(−6.01, 4.65)	59.55 (56.16, 62.77)	61.62 (58.11, 64.95)	55.98 (52.08, 60.17)	−1.52 (−9.51, 7.17)
Sex														
Male	733	6.84	−3.95	5.42	−4.45	396	3.70	0.27	2.91	−0.80	57.45	59.65	53.15	−2.21 (−9.17, 5.28)
			(−14.34, 7.70)		(−14.87, 7.24)			(−5.04, 5.88)		(−6.06, 4.74)	(53.46, 61.23)	(55.51, 63.58)	(48.65, 58.06)	
Female	263	2.41	−4.67	1.83	−5.15^*^	117	1.07	0.33	0.80	−1.12	65.11	66.79	62.27	1.82 (−8.75, 13.62)
			(−9.40, 0.30)		(−9.99, −0.04)			(−11.26, 13.43)		(−12.59, 11.86)	(58.49, 70.94)	(60.00, 72.77)	(55.35, 70.04)	
Region														
Urban	693	4.72	−5.08^d^	3.66	−4.98^e^	346	2.35	3.15	1.83	1.86	61.32	63.43	57.16	0.61 (−9.56, 11.91)
			(−10.6, 0.78)		(−10.98, 1.42)			(−5.41, 12.49)		(−6.39, 10.83)	(57.20, 65.18)	(59.17, 67.42)	(52.35, 62.41)	
Rural	303	2.41	−4.41	3.44	−4.87	167	2.41	−3.47	1.83	−4.02	55.80	57.73	50.92	−1.59 (−9.78, 7.35)
			(−9.81, 1.32)		(−10.52, 1.14)			(−9.54, 3.02)		(−9.73, 2.06)	(49.72, 61.45)	(51.44, 63.57)	(44.99, 57.62)	
Age^a^														
0–44	282	1.96	−3.90^f^ (−13.13, 6.31)	NA		99	0.69	1.82 (−14.95, 21.88)	NA		72.98 (67.14, 77.96)	73.41 (67.53, 78.41)	NA	2.82 (−0.01, 5.74)
45–54	282	9.11	−7.10^*^ (−11.21, −2.81)	NA		145	4.68	1.10 (−6.58, 9.40)	NA		62.92 (56.72, 68.48)	64.19 (57.86, 69.86)	NA	−0.41 (−4.73, 4.11)
55–64	246	11.51	−5.22 (−11.76, 1.81)	NA		133	6.22	0.17 (−6.04, 6.78)	NA		56.15 (48.66, 62.97)	58.66 (50.84, 65.78)	NA	4.13 (−1.96, 10.61)
65+	186	9.50	−3.29 (−8.48, 2.19)	NA		136	6.95	−2.76 (−11.45, 6.79)	NA		33.10 (25.34, 41.04)	38.00 (29.10, 47.12)	NA	−1.26 (−17.33, 17.93)

All rates are expressed per 100,000

*Abbreviations*: *CIR* Crude incidence rate, *ASIR* Age-standardized incidence rate, *CMR* Crude mortality rate, *ASMR* Age-standardized mortality rate, *OS* Observed survival rate, *RS* Relative survival rate, *ARS* Age-standardized relative survival rate,* AAPC* Average annual percent change, *CI* Confidence interval

*P value<0.05

^a^Within each age group, the weights are consistent, making it impossible to further standardize the rates

^b^The AAPC was calculated based on ARS, with the exception of the age subgroup, where RS was used

^c^In total, the APC of CIR was −9.54 (95% CI: −17.57, −0.74; P=0.039) from 2014 to 2020

^d^In urban population, the APC of CIR was −13.07 (95% CI: −20.83, −4.54; P=0.012) from 2015 to 2020

^e^In urban population, the APC of ASIR was −10.61(95% CI: −16.81, −3.96; P=0.010) from 2014 to 2020

^f^In total population between 0–44 years old group, the APC of CIR was −12.97 (95% CI:−22.14, −2.73; P=0.024) from 2014 to 2020

A significant decreasing trend in ASIR of NPC was observed in total population and female population, with AAPCs of −5.48 (95% CI: −9.25, −1.54; *P* < 0.05) and − 5.15 (95% CI: −9.99, −0.04; *P* < 0.05), respectively. Although AAPCs for CIR and ASIR exhibited a declining trend among males and within both urban and rural populations, none reached statistical significance. The most significant decrease in NPC incidence was in the 45–54 age group, with an AAPC of −7.10 (95% CI: −11.21, −2.81; *P* < 0.05).

Further analysis of APC was further conducted to characterize the trend. The decreasing trend of CIR was significant in the period from 2014 to 2020, with the APC of −9.54 (95%CI: −17.57, −0.74; *P* = 0.039), especially in population living in the urban and people aged 0–44 years.

### Mortality trends analysis

Throughout the study period, there were 513 deaths among patients with NPC. The CMR and the ASMR were 2.37 per 100,000 and 1.83 per 100,000, respectively. The ASMR was significantly higher in males than in females (2.91 per 100,000 vs. 0.80 per 100,000), while urban and rural residents showed similar rates (1.83 per 100,000). In all age groups, the CMR increases with age and reached 6.95 per 100,000 for those aged 65 and above. The cumulative mortality rate from 0 to 74 years old was 0.22%, whereas the mortality rate over the truncated age range of 35–64 years was 0.69 per 100,000 (Table S2). The age-standardized YLL rate of NPC was 65.10 per 100,000 for both sexes combined, with rates of 103.85 in males and 28.08 in females (Fig. 1).

NPC mortality was observed with a rapid rise from 2011 to 2015 and a slight decline from 2016 to 2020. However, the overall trend in ASMR for NPC among the total population did not reach statistical significance, with an AAPC of −0.82% (95% CI: −6.01, 4.65). Specifically, ASMRs showed a declining trend among males, females, and rural residents, whereas a slight increase was observed in urban residents; however, none of these trends reached statistical significance within their respective subgroups (Table 1; Fig. 1). Age-standardized YLL rate of NPC showed similar trend as the ASMR from 2011 to 2020 (Fig. 1).

### The survival rate trend analysis

The 5-year OS, RS, and ARS in Xiamen residents with NPC were 59.55% (95%CI: 56.16, 62.77), 61.62% (95%CI: 58.11, 64.95), 55.98% (95%CI: 52.08, 60.17) (Table 1). Between 2011 and 2020, in Xiamen, the 5-year ARS saw an improvement from 50.48% (95% CI: 44.88, 56.76) in the period of 2011 to 2015, to 59.01% (95% CI: 53.44, 65.17) between 2016 and 2020. As shown in Table 1, Table S3, and Figure S1, the 5-year ARS was lower in males compared to females (53.15% vs. 62.27%), and is also lower in rural areas compared to urban areas (50.92% vs. 57.16%). The highest 5-year RS rate was observed in the youngest age group (0–44 years) at 73.41%, followed by a gradual decline to 64.19% in the 45–54 age group and 58.66% in the 55–64 age group, before plummeting to 38.00% in individuals aged 65 years and older (Table 1; Fig. 1).

However, from 2011 to 2020, the AAPC in the 5-year ARS showed no statistical significance in either the total population or any of the subgroups, including sex, region, and age groups (Table 1; Fig. 1).

## Discussion

This study comprehensively investigated the incidence, mortality, YLL and survival rate of NPC in Xiamen, China, from 2011 to 2020. Age-standardized incidence, mortality and YLL rates were 3.58/100,000, 1.83/100,000 and 65.10/100,000 respectively. The 5-year ARS in Xiamen residents with NPC was 55.98% (95%CI: 52.08, 60.17). Our study showed that the ASIR of NPC in Xiamen decreased steadily since 2011 by the AAPC of 5.48%. The overall trends in NPC mortality and survival rate were not statistically significant, the trends in mortality rate and YYL rate were similar, neither was significant. The 5-year ARS exhibited a modest increase from 50.48% (95% CI: 44.88, 56.76) during the period from 2011 to 2015, to 59.01% (95% CI: 53.44, 65.17) in the subsequent period from 2016 to 2020.

The encouraging incidence reduction seen in this study was similar to those observed in other regions with high NPC incidence rates. In urban Guangzhou, a consistent decreasing trend in ASIR was observed with an AAPC of −3.26% in males and − 5.74% in females from 2000 to 2011 [7]. In Hong Kong, from 1991 to 2020, the CIR and ASIR of NPC also decreased significantly [9]. However, in other regions of China, a different trend of NPC incidence was shown. The incidence rates of NPC in Shanghai, Wuhan, Zhongshan, Sihui, and Cangwu were stable over a long period [8, 10–12]. Notably, several studies have indicated an increase in both ASIR and CIR for the last three decades in China [6, 13]. These observations highlight the geographically specific pattern in the epidemiology of NPC, even within the same country, and underscore the necessity for a localized approach in both the prevention and intervention of the disease.

The occurrence of NPC may be attributed to multiple factors, including genetic predisposition, dietary habits and lifestyle, and their complex interactions [1, 14]. The decreasing trend in NPC incidence observed in Xiamen may be related to changes in the factors related to lifestyle and/or environment, such as smoking, and the consumption of alcohol and salted fish, and exposure to carcinogens.

Previous studies reported that the ASMRs in both sexes’ groups and the CMR in females have decreased from 1990 to 2019 in China [6, 15]. Furthermore, previous studies reported that the majority of provinces in China experienced a reduction in YLL rates of NPC, with the magnitude of the decline ranging from 5.9% to 65.1% from 2005 to 2020 [15]. However, in Xiamen, ASMR and YLL rates showed only a modest decline from 2011 to 2020. The mortality and YLL levels remained relatively high, warranting attention as an important public health problem. A higher ASMR (2.91 per 100,000) was observed among males in Xiamen, compared with females (0.80 per 100,000), which is consistent with previous studies [16]. The lower risk of developing and dying from NPC observed in females across all races is an intriguing phenomenon. Potential explanations include genetic differences related to the X chromosome or estrogen sex hormones, or possibly both [17]. Additionally, males may face higher exposure to occupational hazards, indulge in smoking more frequently, consume alcohol in greater quantities, and have less awareness of their health status.

Huang et al. reported on contemporary survival outcomes in Taiwan [18], indicating a five-year overall survival rate of 65.2%. The study found male gender, advanced age, residence in eastern Taiwan, low income, and receiving chemotherapy alone as independent predictors of poorer overall survival rate. It is indeed a common finding that female patients have a more favorable prognosis, and is consistent with our study [9, 19]. In our study, as age increases, the 5-year RS for NPC decreases. Additionally, the survival rate of NPC in urban areas exceeds that in rural areas, and cases diagnosed between 2016 and 2020 exhibiting higher survival rates compared to those diagnosed between 2011 and 2015. This improvement in survival rates may be attributed to advancements and increased investment in medical care and technologies related to screening and treatment. However, the equality of healthcare resources and capabilities between urban and rural areas still needs improvement. Implementing policies that enhance funding for rural healthcare, incentivize medical professionals, and invest in telemedicine could help address these inequities.

In our study, the 5-year ARS in Xiamen residents with NPC was 55.98% (95%CI: 52.08, 60.17). Previous studies have shown that patients diagnosed with early-stage NPC, specifically stages I and II, exhibit favorable treatment outcomes, achieving a 5-year survival rate of up to 84%−90%. This rate is markedly higher compared to that of patients diagnosed with late stage (stages III and IV: 30%−55%) NPC [20, 21]. In China, several targeted screening initiatives have been implemented in high-incidence areas, involving annual detection of EBNA1-IgA and VCA-IgA and subsequent nasopharyngoscopy for high-risk individuals. In Guangdong Province, an NPC screening program has been in place since 1986, enrolling 98,180 high-risk residents [22]. The 5-year survival rate for screened individuals reached 79.87%, significantly outperforming the survival rate (58.43%) observed in hospitalized cases during the same period [8]. A prospective, cluster-randomized, controlled trial for NPC screening conducted in southern China (Zhongshan and Sihui) beginning in 2008 indicated a significant reduction in NPC-specific mortality among the screening participants [17]. Screening could be one of the main measures in decreasing mortality of NPC, by promoting early diagnosis and treatment. Circulating serological markers of EBV infection (specifically EBNA1-IgA, VCA-IgA) and plasma EBV DNA have shown the most compelling evidence of utility in NPC screening [23, 24]. A recent study by Li et al. suggested that a new candidate serological biomarker of EBV infection, an anti-BNLF2b antibody, might also be an effective screening tool [25]. However, compared to high-risk regions in Guangdong and Guangxi, where screening interventions have been promoted, the decline in ASMR in Xiamen over recent years has not been significant [15]. Moreover, the OS in Xiamen (59.55%) is lower than the previously reported rate in the high-risk region of Sihui (69.8%). Without screening, the majority (60–70%) of NPC patients are diagnosed with locoregionally advanced disease due to the intrinsic invasiveness and asymptomatic nature of the disease [26]. It indicates that screening is also important even in intermediate- and low- risk regions, based on individual risk. Future research should focus on assessing the cost-effectiveness of screening programs in regions with diverse risk levels, aimed at devising more targeted and effective prevention strategies [27–31].

A significant limitation of this study is that the data were exclusively collected from Xiamen, which may limit the generalizability of the findings to other regions or populations. Additionally, we supplemented our data with cancer cases confirmed through the Xiamen Death Registry. However, these cases typically do not include comprehensive clinical details, such as stage at diagnosis, EBV DNA viral load, and treatment information, which limits more in-depth analyses. We did not gather data on individual-level risk factors such as smoking and alcohol consumption, which may limit the extent to which causal interpretations of the observed trends can be drawn. Finally, the observation period of 10 years (2011–2020), while sufficient for preliminary trend assessment, may constrain the robustness of long-term trend analyses. Future studies incorporating multi-regional data, comprehensive clinical information, and extended timeframes will be essential to validate and extend the present results.

Overall, this study revealed a steady decline in NPC incidence and insignificant decreasing mortality trend in Xiamen. Further enhancements in tailoring the region- and population- specific NPC control strategy could be beneficial to consolidate these positive trends.

## Supplementary Information

Supplementary Material 1

## Data Availability

The study data are available for academic purposes upon reasonable request. To gain access, please contact corresponding author Yilan Lin (linyl254@163.com) for further details.

## Abbreviations

NPC: nasopharyngeal carcinoma
YLL: years of life lost
ARS: age-standardized relative survival rate
IARC: International Agency for Research on Cancer
ASIR: age-standardized incidence rates
ASMR: age-standardized mortality rate
CMR: crude mortality rate
CIR: crude incidence rate
DCO%: death certification only
MV%: proportion of morphological verification
M/I: mortality-to-incidence ratio
APC: annual percent changes
AAPC: average annual percent changes
GBD 2019: Global Burden of Disease Study 2019
OS: observed survival rate
RS: relative survival rate
ARS: age-standardized relative survival rate
ICCS: the International Cancer Survival Standards
