# Supplementary material for: The nasopharyngeal carcinoma in Xiamen China from 2011 to 2020: a population-based linkage study
Source: BMC Cancer. 2025 Dec 8;26:72. doi: 10.1186/s12885-025-15394-0 (PMC12802225; doi:10.1186/s12885-025-15394-0)
Supplement: Supplementary file 1 — Supplementary Material 1 [file 12885_2025_15394_MOESM1_ESM.docx]

**Supplementary File**

**Table S1. Annual cumulative incidence rates and truncated rates of nasopharyngeal cancer by gender and region from 2011 to 2020**

| **Year** | **Cumulative rate**  **（0-74 years old, %）** | | | | | **Truncated rate**  **(35-64 years old, 1/10^5^）** | | | | |
| --- | --- | --- | --- | --- | --- | --- | --- | --- | --- | --- |
|  | **sex** | | **region** | | Total | **sex** | | **region** | | Total |
|  | Male | Female | Urban | Rural |  | Male | Female | Urban | Rural |  |
| 2011 | 0.57 | 0.27 | 0.44 | 0.37 | 0.42 | 12.32 | 5.20 | 8.95 | 8.43 | 8.74 |
| 2012 | 0.79 | 0.26 | 0.52 | 0.52 | 0.52 | 16.79 | 6.35 | 11.59 | 11.41 | 11.49 |
| 2013 | 0.74 | 0.19 | 0.45 | 0.47 | 0.46 | 15.56 | 3.31 | 10.12 | 7.95 | 9.28 |
| 2014 | 0.75 | 0.23 | 0.52 | 0.4 | 0.48 | 15.97 | 4.04 | 10.02 | 9.64 | 9.93 |
| 2015 | 0.69 | 0.22 | 0.49 | 0.39 | 0.45 | 12.00 | 3.64 | 9.61 | 3.88 | 7.75 |
| 2016 | 0.79 | 0.19 | 0.5 | 0.44 | 0.48 | 16.63 | 4.16 | 11.09 | 8.67 | 10.25 |
| 2017 | 0.46 | 0.15 | 0.34 | 0.23 | 0.30 | 9.74 | 3.01 | 6.89 | 5.11 | 6.31 |
| 2018 | 0.40 | 0.28 | 0.32 | 0.37 | 0.34 | 8.89 | 5.86 | 7.21 | 7.75 | 7.38 |
| 2019 | 0.60 | 0.13 | 0.33 | 0.42 | 0.36 | 12.01 | 2.16 | 6.23 | 8.84 | 6.98 |
| 2020 | 0.39 | 0.13 | 0.23 | 0.29 | 0.25 | 7.36 | 2.51 | 4.78 | 4.83 | 4.83 |
| Total | 0.60 | 0.20 | 0.4 | 0.39 | 0.40 | 12.45 | 3.94 | 8.39 | 7.59 | 8.11 |
| AAPC | -5.74^*^  (-10.94, -0.23) | -6.40^*^  (-11.55, -0.96) | -6.67^*^  (-12.31, -0.67) | -3.82  (-8.99, 1.65) | -5.94^*^  (-9.66, -2.06) | -6.41^*^  (-11.41, -1.14) | -7.01  (-13.7, 0.19) | -7.72^*^  (-12.84, -2.31) | -4.75  (-12.43, 3.6) | -6.48^*^  (-10.46, -2.32) |

**P* values<0.05

**Table S2. Annual cumulative mortality rates and truncated rates of nasopharyngeal cancer by gender and region from 2011 to 2020**

| **Year** | **Cumulative rate**  **（0-74 years old, %）** | | | | | **Truncated rate**  **(35-64 years old, 1/10^5^）** | | | | |
| --- | --- | --- | --- | --- | --- | --- | --- | --- | --- | --- |
|  | **sex** | | **region** | | Total | **sex** | | **region** | | Total |
|  | Male | Female | Urban | Rural |  | Male | Female | Urban | Rural |  |
| 2011 | 0.37 | 0.11 | 0.22 | 0.28 | 0.24 | 7.00 | 1.73 | 3.38 | 6.35 | 4.42 |
| 2012 | 0.29 | 0.08 | 0.14 | 0.28 | 0.19 | 4.07 | 1.15 | 2.48 | 2.96 | 2.64 |
| 2013 | 0.36 | 0.07 | 0.23 | 0.19 | 0.21 | 6.86 | 0.70 | 4.55 | 2.53 | 3.81 |
| 2014 | 0.34 | 0.14 | 0.22 | 0.27 | 0.24 | 6.69 | 2.36 | 3.56 | 6.42 | 4.58 |
| 2015 | 0.54 | 0.14 | 0.36 | 0.28 | 0.34 | 9.12 | 2.72 | 5.96 | 5.51 | 5.86 |
| 2016 | 0.29 | 0.10 | 0.21 | 0.16 | 0.19 | 7.02 | 2.10 | 5.13 | 3.46 | 4.57 |
| 2017 | 0.37 | 0.02 | 0.19 | 0.19 | 0.19 | 5.85 | 0.40 | 3.44 | 2.42 | 3.11 |
| 2018 | 0.32 | 0.13 | 0.2 | 0.26 | 0.22 | 6.33 | 1.88 | 4.04 | 4.4 | 4.18 |
| 2019 | 0.28 | 0.07 | 0.19 | 0.13 | 0.17 | 5.41 | 1.17 | 3.22 | 3.87 | 3.38 |
| 2020 | 0.31 | 0.10 | 0.21 | 0.19 | 0.20 | 6.73 | 2.49 | 4.59 | 4.83 | 4.61 |
| Total | 0.34 | 0.09 | 0.22 | 0.22 | 0.22 | 6.49 | 1.68 | 4.06 | 4.24 | 4.11 |
| AAPC | -1.68  (-6.46, 3.35) | -2.91  (-16.67, 13.12) | 0.03  (-6.06, 6.51) | -5.25  (-10.75, 0.58) | -2.08  (-6.82, 2.9) | 0.35  (-5.13, 6.15) | 1.68  (-13.88, 20.06) | 2.29  (-4.3, 9.33) | -0.73  (-9.86, 9.32) | 0.69  (-5.49, 7.28) |

**Table S3. The survival rates of nasopharyngeal cancer in different year in Xiamen city from 2011 to 2020**

| Year | 1-year survival rate | | | 3-year survival rate | | | 5-year survival rate | | | 10-year survival rate | | |
| --- | --- | --- | --- | --- | --- | --- | --- | --- | --- | --- | --- | --- |
|  | OS  (95%CI) | RS  (95%CI) | ARS  (95%CI) | OS  (95%CI) | RS  (95%CI) | ARS  (95%CI) | OS  (95%CI) | RS  (95%CI) | ARS  (95%CI) | OS  (95%CI) | RS  (95%CI) | ARS  (95%CI) |
| 2011 | 77.96  (66.61, 85.86) | 78.46  (67.03, 86.4) | 78.96  (68.39, 91.16) | 59.87  (48.38, 69.60) | 61.17  (49.43, 71.11) | 59.84  (47.78, 74.94) | 50.96  (35.69, 64.33) | 53.11  (37.2, 67.05) | 47.47  (33.7, 66.85) | 16.63  (0.21, 59.31) | 18.16  (0.23, 64.74) | 17.63  (11.84, 26.25) |
| 2012 | 89.78  (81.28, 94.54) | 90.55  (81.98, 95.36) | 78.04  (70.67, 86.17) | 74.38  (62.56, 82.96) | 76.00  (63.92, 84.78) | 65.04  (55.84, 75.75) | 65.67  (49.04, 78.03) | 68.44  (51.11, 81.31) | 54.74  (41.73, 71.79) | 65.67  (49.04, 78.03) | 71.74  (53.57, 85.23) | 31.34  (21.65, 45.36) |
| 2013 | 86.21  (77.00, 91.92) | 86.85  (77.58, 92.60) | 87.99  (81.11, 95.45) | 70.71  (58.94, 79.68) | 72.18  (60.15, 81.33) | 76.89  (67.35, 87.78) | 62.72  (50.71, 72.59) | 65.06  (52.6, 75.29) | 73.38  (62.76, 85.78) | 42.11  (11.28, 70.98) | 45.98  (12.32, 77.5) | 32.28  (26.64, 39.12) |
| 2014 | 89.03  (81.08, 93.77) | 89.62  (81.61, 94.38) | 79.99  (73.71, 86.8) | 65.74  (54.90, 74.56) | 67.16  (56.09, 76.18) | 58.55  (49.48, 69.28) | 55.61  (44.13, 65.65) | 57.61  (45.71, 68.00) | 51.72  (41.81, 63.98) | 23.57  (1.86, 59.22) | 25.92  (2.04, 65.11) | 11.78  (7.57, 18.32) |
| 2015 | 80.19  (71.02, 86.72) | 80.81  (71.57, 87.39) | 73.08  (63.85, 83.65) | 59.15  (49.00, 67.94) | 60.36  (50.00, 69.32) | 56.06  (46.02, 68.29) | 43.97  (33.69, 53.78) | 45.38  (34.76, 55.50) | 36.36  (28.80, 45.89) | 36.83  (27.16, 46.51) | 40.16  (29.61, 50.72) | 24.43  (17.99, 33.16) |
| 2016 | 78.30  (69.20, 85.00) | 79.00  (69.82, 85.76) | 75.22  (67.49, 83.83) | 65.73  (55.82, 73.94) | 67.02  (56.92, 75.39) | 60.24  (52.01, 69.77) | 62.14  (51.95, 70.77) | 64.06  (53.56, 72.96) | 58.73  (50.15, 68.77) | 38.47  (19.77, 56.96) | 41.98  (21.57, 62.15) | 32.93  (25.62, 42.33) |
| 2017 | 80.88  (71.70, 87.34) | 81.64  (72.38, 88.16) | 78.40  (71.29, 86.22) | 66.12  (56, 74.45) | 67.55  (57.21, 76.06) | 64.96  (56.13, 75.18) | 63.33  (53.17, 71.86) | 65.43  (54.93, 74.24) | 59.05  (50.70, 68.77) | 49.06  (34.08, 62.43) | 53.16  (36.93, 67.65) | 47.47  (36.1, 62.43) |
| 2018 | 88.65  (79.34, 93.93) | 89.18  (79.81, 94.48) | 90.94  (84.51, 97.86) | 70.75  (59.92, 79.15) | 72.17  (61.12, 80.75) | 77.97  (69.76, 87.13) | 60.6  (49.76, 69.81) | 62.52  (51.33, 72.02) | 50.51  (42.7, 59.76) | 45.22  (33.99, 55.78) | 48.63  (36.55, 59.99) | 49.55  (32.93, 74.56) |
| 2019 | 93.50  (86.11, 97.03) | 94.09  (86.65, 97.64) | 94.19  (88.63, 100.11) | 81.63  (71.30, 88.54) | 83.26  (72.72, 90.3) | 86.23  (78.22, 95.06) | 69.56  (58.33, 78.31) | 71.84  (60.24, 80.88) | 62.93  (50.29, 78.74) | 59.69  (47.52, 69.92) | 63.68  (50.7, 74.59) | 55.56  (41.51, 74.37) |
| 2020 | 88.35  (79.44, 93.55) | 88.99  (80.01, 94.23) | 87.66  (79.60, 96.53) | 71.46  (60.73, 79.75) | 72.85  (61.9, 81.29) | 66.72  (58.57, 76.00) | 60.15  (49.12, 69.52) | 62.04  (50.67, 71.71) | 57.30  (48.07, 68.29) | 48.66  (37.47, 58.95) | 51.68  (39.8, 62.6) | 51.8  (41.86, 64.11) |
| Total | 85.12  (82.65, 87.27) | 85.81  (83.32, 87.98) | 80.56  (77.57, 83.67) | 68.42  (65.26, 71.35) | 69.88  (66.66, 72.89) | 64.08  (60.47, 67.91) | 59.55  (56.16, 62.77) | 61.62  (58.11, 64.95) | 55.98  (52.08, 60.17) | 45.66  (41.34, 49.87) | 49.25  (44.59, 53.79) | 43.03  (37.75, 49.04) |

Abbreviations: OS, observed survival rate; RS, relative survival rate; ARS, age-standardized relative survival rate. AAPC, average annual percent change; CI, confidence interval.


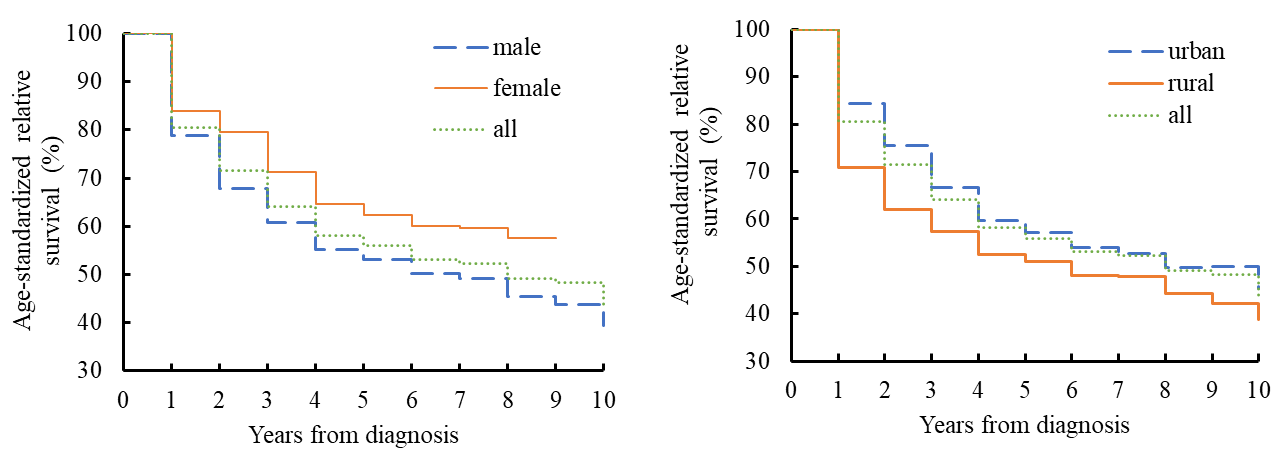


**Figure S1. Age-standardized relative survival by years from diagnosis in Xiamen.**
